# A NOVEL PREOPERATIVE CALCULATOR FOR PERIPROSTHETIC JOINT INFECTION FOLLOWING TOTAL JOINT ARTHROPLASTY BASED OFF THE MARCQI DATABASE

**DOI:** 10.51894/001c.122804

**Published:** 2024-08-30

**Authors:** Daniel Gerow, Huiyong Zheng, Richard Hughes, Kory Johnson, Brian Hallstrom

**Affiliations:** 1 Health West- Orthopedic Surgery University of Michigan

## 06

### BACKGROUND

Total joint arthroplasty has become one of the most successful surgeries in regard to quality of life and cost effectiveness. Periprosthetic joint infections can have catastrophic effects on a patient’s surgical outcome, quality of life, and function. Shared decision making can help patients reduce their risk by changing modifiable risk factors.

### OBJECTIVES

We set out to create an infection risk prediction model for total joint arthroplasty of hips and knees.

### METHODS

Logistic regression was used to construct an infection risk prediction model for primary total joint arthroplasty of hips and knees using data from the Michigan Arthroplasty Registry Collaborative Quality Initiative (MARCQI) dataset, which includes 140,884 total knee arthroplasties (TKA) and 82,110 total hip arthroplasties (THA). The resulting model was deployed using a web-based interface that allows surgeons to counsel their patients in the clinic by showing them in real time how adjusting modifiable risk factors will affect their overall risk of infection.

### RESULTS

To our knowledge, this study is novel in finding preoperative opioid use and use of assistive devices to be predictive of infection after primary TKA and THA. Modifiable risk factors that were found to significantly affect risk included BMI, smoking, and preoperative narcotic use. Nonmodifiable risk factors included sex, marital status, race, type of payer, preoperative use of assistive devices, and annual surgeon volume < 100 cases per year.

### CONCLUSIONS

While some of these risk factors are non-modifiable, others can be addressed and optimized prior to surgery. Surgeons must be equipped with infection risk information to accurately counsel patients preoperatively, while also using data to drive patient selection.

